# Patient Sexual Orientation and Gender Identity Information Practices in Oncology

**DOI:** 10.1001/jamanetworkopen.2025.16941

**Published:** 2025-06-23

**Authors:** Mandi L. Pratt-Chapman, Megan A. Mullins, Beck O. Gold, Milena E. Insalaco, Dana Rosenberg, Edward J. Miech, Charles Kamen

**Affiliations:** 1 Department of Medicine, GW Cancer Center, The George Washington University School of Medicine and Health Sciences, Washington, DC; 2 Department of Health Economics, Systems, and Policy, O’Donnell School of Public Health, UT Southwestern Medical Center, Dallas, Texas; 3 Division of Supportive Care in Cancer, Department of Surgery, University of Rochester Medical Center, Rochester, New York; 4 Department of Emergency Medicine, Indiana University School of Medicine, Indianapolis

## Abstract

**Importance:**

Sexual orientation and gender identity (SOGI) are rarely collected in clinical practice despite national calls to do so.

**Objective:**

To explore what facilitators are associated with collection of SOGI data in oncology practice.

**Design, Setting, and Participants:**

In this qualitative study with purposive sampling for greatest heterogeneity, semistructured interviews across 23 institutions were conducted in oncology practices in diverse geopolitical regions in the US from September 1, 2022, to August 31, 2023.

**Main Outcomes and Measures:**

Interviews were transcribed, double coded using deductive coding based on the Consolidation Framework for Implementation Research, and inductively coded based on emergent themes.

**Results:**

Of 23 cancer centers, most were academic cancer centers (14 [61%]) and located in urban settings (17 [74%]); 7 sites (30%) sites systematically collected SOGI data, 11 (48%) partially collected SOGI data, and 5 (22%) did not collect SOGI data. A total of 62 interviews were conducted. Mean (SD) participant age was 46 (11) years; 26 (42%) identified as female. Facilitators associated with collection of SOGI data included external mandates, forced workflows in the electronic medical record, structured data fields, perception that SOGI data were relevant to clinical practice, and leadership support. Among sites that partially collected SOGI data, existence of champions, training for SOGI data collection, and perception of the relevance of SOGI to cancer care were facilitators associated with collection. Sites that systematically collected SOGI data offered more rigorous training on the importance of SOGI data collection for use in practice. Sites that did not collect SOGI data indicated that a mandate or top-down leadership requirement would likely be needed to catalyze SOGI data collection.

**Conclusions and Relevance:**

This qualitative study of implementation factors associated with SOGI data collection in outpatient oncology practices found that systematic SOGI data collection occurred in a few centers. Factors associated with SOGI data collection were multifaceted, involving both external mandates and internal factors. Despite the collection of SOGI data, their clinical use varied, suggesting a need for ongoing clinician education and development of strategies to improve the integration of SOGI data into patient care.

## Key Points

**Question:**

What facilitators are associated with collection of sexual orientation and gender identity (SOGI) data in oncology practice?

**Findings:**

In this qualitative study, 62 individuals interviewed at 23 cancer centers reported that external mandates, forced workflows in the electronic medical record, structured data fields, perception that SOGI data were relevant to clinical practice, and leadership support were associated with collection of SOGI data. Sites that did not collect these data indicated that a mandate or top-down leadership would be required to advance future data collection.

**Meaning:**

This study suggests that mandates, forced workflows, structured data fields, and leadership support are facilitators associated with systematic data collection of new and potentially controversial demographic fields.

## Teaser

This qualitative study explores what facilitators are associated with collection of sexual orientation and gender identity data in oncology practice.

## Introduction

In 2015, the Centers for Medicare & Medicaid Services required electronic health record (EHR) systems to be able to document patients’ sexual orientation and gender identity (SOGI) to align with stage 3 of Meaningful Use.^1^ In 2017 and 2023, the American Society of Clinical Oncology (ASCO) called for SOGI data collection to address the needs of sexual and gender minority (SGM) people affected by cancer, noting disparities at every stage of the continuum.^2^

In 2011 and 2020, the National Academies of Sciences, Engineering, and Medicine (NASEM) published reports on the health of SGM people, and in 2022, NASEM reported a summary of evidence on SOGI data collection items used in health care.^3^ A National Cancer Institute–funded working group subsequently published consensus-based recommendations for SOGI item wording.^4^ Prior research found that collection of SOGI data was associated with leadership support, resources, and belief in the importance of collecting SOGI data.^5^ The present study sought to explore these factors in greater detail from a qualitative perspective.

## Methods

### Study Population

From September 1, 2022, to August 31, 2023, doctorally prepared qualitative researchers (M.L.P.-C., M.A.M., and C.K.) conducted 62 interviews in 23 oncology practices with diverse geopolitical locations, academic or nonacademic affiliations, patient mixes, sizes, and settings until data saturation was reached. The George Washington University, UT Southwestern Medical Center, and University of Rochester institutional review boards approved this study. The purpose of the study was reviewed by the interviewer, and oral informed consent from all participants was provided before proceeding with or recording interviews. This study followed the Consolidated Criteria for Reporting Qualitative Research (COREQ) reporting guideline.

### Recruitment

An email was sent to 2325 ASCO members requesting participants to complete a REDCap, version 15.0.10 (Vanderbilt University) screening questionnaire that queried respondents on geographic location, ability to recruit a staff member and an administrator who worked with demographic data from their cancer center, and practice setting (private vs academic). Twenty-three unique screening questionnaires were received from individuals representing 22 institutions. Ten institutions were recruited for interviews. Seven additional institutions were added from a separate recent study^6^ that asked similar questions. ASCO membership lists were reviewed to actively recruit 6 additional centers to maximize diversity of geographic representation and SOGI data collection practices based on comparison with already recruited sites. Key stakeholders at each practice (range, 1-4) were interviewed separately. Eight individuals dropped out of the study due to competing time demands. No prior relationships between study team members and participants existed.

### Data Collection

A semistructured guide queried stakeholders about the degree to which the practice collected SOGI data, along with quality and completeness of data, implementation constructs from the Consolidated Framework for Implementation Research (CFIR), and recommendations for improved data collection (eAppendix in Supplement 1). Interviews were approximately 60 minutes. Zoom (Zoom Video Communications) recordings were stored on The George Washington University Box system, transcribed via Zoom, and manually checked for accuracy. Summaries of major findings of each interview were emailed to each participant to confirm accuracy. Reflective memos were noted by each interviewer.

### Data Analysis

Underpinned by content analysis theory and using a combination of Dedoose, version 9.2.012 (Sociocultural Research Consultants, LLC), NVivo, versions 1.7.2 and 14.23.4 (Lumivero), and Google Sheets (Google LLC) software, interviews were double coded using a mix of deductive coding based on major CFIR constructs and inductive coding based on identification of new themes (eg, responses not anticipated based on the interview guide). Four team members (M.L.P.-C., B.O.G., M.E.I., and D.R.) coded 6 transcripts in common to create a codebook of prevalent CFIR constructs. Example quotations of constructs were mapped onto a shared Google spreadsheet, and consensus was obtained through discussion. When additional constructs emerged in the data, these were added to the codebook, discussed for consensus, and coded with exemplar quotations.

#### Site Characteristics

With assistance from ASCO staff, we purposively selected ASCO members from cancer centers that varied based on size of center, geography, and self-reported collection of SOGI (Table 1). We considered a hospital or cancer center to be academic if it had a partnership with a medical school and served as a site for medical education; otherwise, it was considered to be nonacademic. Settings described the location of the main campus of the cancer center based on urban, suburban, or rural selection by respondents. For geographic area, we assigned states based on the US Census designation of Northeast, Midwest, South, or West.^7^ Practice type was self-selected from physician owned, practice owned by hospital or health system, or other. Institutional characteristics including accreditations and standards were verified with hospital websites, accreditation sources, available online information, or self-reported by interviewees.^8,9,10,11,12^ Because Medicaid expansion is often a proxy for geopolitical environment, we also verified Medicaid expansion of each institution’s home state through Kaiser Family Foundation’s tool.^13^

**Table 1.** Site Characteristics of Respondents

Characteristic	No. (%) (N = 23)
Prevalence of SOGI data collection (based on analysis)	
Systematically collects SOGI	7 (30)
Sometimes collects SOGI	11 (48)
Does not collect SOGI	5 (22)
Type of cancer center	
Academic cancer center	14 (61)
Nonacademic cancer center	7 (30)
Missing classification	2 (9)
Accreditations and standards^a^	
American Society of Clinical Oncology–Quality Oncology Practice Initiative Certification	10 (44)
American College of Surgeons Commission on Cancer Accreditation	19 (83)
Human Rights Campaign Healthcare Equality Index	13 (57)
National Cancer Institute–Designated Cancer Centers	8 (35)
National Standards for Culturally and Linguistically Appropriate Services	2 (9)
Cancer program setting	
Urban (a town or a city)	17 (74)
Suburban (the outlying district of a city)	4 (17)
Rural	1 (4)
Missing classification	1 (4)
Geographic area	
Northeast	6 (26)
South	5 (22)
Midwest	7 (30)
West	5 (22)
Medicaid expansion	
Yes–before 2019	19 (83)
No	4 (17)
Practice type	
Practice owned by hospital or health system	20 (87)
Physician-owned practice (ie, independent, office-based)	3 (13)

Abbreviation: SOGI, sexual orientation and gender identity.

^a^ Sites could select more than 1 option.

Respondents reported the following individual data through REDCap: gender identity and sexual orientation via open text response, age in years, and role within the cancer center (Table 2). Because we supplemented primary data collection with data collected separately through a similarly designed study^6^ that did not collect demographic variables, we do not have demographic data for 12 transcripts.

**Table 2.** Demographic Characteristics of Respondents

Characteristic	No. (%) (N = 62)
Gender identity	
Cisgender female	1 (2)
Cisgender male	2 (3)
Cisgender woman	1 (2)
Cisgender man	1 (2)
Woman	3 (5)
Man	0
Female	26 (42)
Male	14 (23)
Transgender	0
Nonbinary	0
Use a different term (write out)	0
Prefer not to answer	0
No data collected or no response	14 (23)
Sexual orientation	
Straight or heterosexual	36 (58)
Lesbian or gay	8 (13)
Bisexual	0
Use a different term (write out)	1 (2)
Do not know	0
Prefer not to answer	2 (3)
No data collected	12 (19)
No response	3 (5)
Role^a^	
Administrator	19 (31)
Clinician	20 (32)
Staff	13 (21)
No data collected	12 (19)
Age, y (n = 49)	
Range	28-68
Mean (SD)	46 (11)

^a^ Individuals could select more than 1 option.

#### Site Categorization

After conducting initial analyses, we categorized sites as systematically collecting, partially collecting, or not collecting SOGI data. Systematic data collection was defined as collecting SOGI data on all patients in structured fields. Partial data collection was defined as having a partially implemented plan to collect SOGI data, SOGI data collection by 1 clinician or clinic, or optional documentation by patient self-report. No data collection was defined as not having structured fields to collect SOGI data, intentionally disabling structured fields that would collect SOGI data, or using neither structured fields to collect SOGI data nor patient self-report (Box 1).

Box 1. **Categorization of Sites**

**Systematic SOGI data collection**

Systematic SOGI data collection in structured fields that is reinforced by training, auditing, and/or forced workflows (n=7)
  - Perception that SOGI is relevant to practice
  - Mandated or forced workflows
  - Structured IT fields
  - Leadership support
  - Champions
  - Training

**Nonsystematic SOGI data collection**

Systematic plan that is not yet implemented or patient self-report option in structured fields or only part of the institution (eg, 1 clinic) collects SOGI data (n=11)
  - Evolving perception that SOGI is relevant to practice
  - Setting up supports (eg, training)
  - Some institutional resources (eg, champions, DEI office)
  - Culture of change

**No data collection**

SOGI data reporting not available in structured fields or intentionally disabled or structured SOGI data fields available but not used and no patient self-report option (n=5)
  - Lack of perception that SOGI is important to practice
  - No structured fields for SOGI data, or fields are disabled or not used
  - Cultural pushback

Abbreviations: DEI, diversity, equity, and inclusion; IT, information technology; SOGI, sexual orientation and gender identity.

## Results

### Characteristics of Sites Systematically Collecting SOGI Data

Of the 23 cancer care sites in our sample, most were academic cancer centers (14 [61%]) and located in urban settings (17 [74%]); 7 (30%) had implemented a systematic process to collect SOGI data from patients, 11 (48%) partially collected SOGI data, and 5 (22%) did not collect SOGI data. A total of 62 interviews were conducted. Mean (SD) participant age was 46 (11) years; 26 (42%) identified as female and 14 (23%) as male. Facilitators associated with data collection included having a mandate to collect SOGI data by a government entity, accreditor, or payer; forced workflows that ensured SOGI data collection; structured EHR fields for SOGI data collection; perception that SOGI data were relevant to practice; and leadership support. Other constructs that were sometimes present at sites that systematically collected SOGI data included having strong clinical and/or administrative champions, reinforcing data collection through training, and reinforcing data collection through public and clinician education.

The presence of a mandate was important for several sites collecting SOGI data systematically, but the level of the mandate differed. One interviewee (academic; Northeast region) indicated:

It’s also the state environment… I think in health care in general in [state name] has been like, you have to start collecting this data… Apparently that’s coming. If you’re getting state funds, I think that might already be in place.

In another institution, a leadership mandate ensured data collection through structured workflows (ie, SOGI data needed to be collected to continue patient registration in the EHR). One interviewee (academic; Northeast) said:

This was taken on by the cancer center as one of their DEI [diversity, equity, inclusion] targets, so it was a mandate from the cancer center to collect [SOGI data], which was a huge aspect of pushing this forward for that 90% level of completion.

Another interviewee (nonacademic; Northeast) reported that they were required to collect SOGI data when they joined a larger academic institution. Perception of pressure from an accrediting or quality assurance body was an external motivator. An interviewee (academic; Northeast) indicated it was “helpful [that] some of the pharmaceutical companies [started] collecting [SOGI data] as part of their clinical trials.” One respondent (academic; Midwest) mentioned tracking Centers for Medicare & Medicaid Services requirements for payment and another respondent (academic; Northeast) referenced ASCO’s accreditation standards regarding data collection equity, including SOGI.

In contrast, respondents at sites where SOGI data were not being collected said that mandates by payers or accrediting organizations would catalyze data collection. One respondent (academic; Midwest) said, “I mean, anything that’s mandated we would do.” Another respondent (nonacademic; South) indicated, “If ASCO [Quality Oncology Practice Initiative] came out and said this is something that’s one of our points of emphasis, we would probably end up [collecting SOGI data].” One site (academic; Northeast) partially collecting SOGI data had begun to implement data collection based on a state mandate but then slowed down implementation when the state delayed timing of the data collection requirement.

Among the centers without a mandate that were still collecting SOGI data, 1 site (academic; Midwest) reported having a forced workflow with hard stops in the EHR: “That’s what they have to do next. You know, social determinants of health, they have to do the SOGI tool, we do the COST-FACIT [Comprehensive Score for Financial Toxicity–Functional Assessment of Chronic Illness Therapy].” There was no way to proceed with scheduling until these data were completed.

A few centers had procedural redundancies to optimize completeness of data collection. For example, 1 center (academic; Midwest) relied primarily on data flowing from primary care to oncology, but reinforced SOGI data collection in oncology with self-report through the MyChart (Epic Systems) portal and paper intake forms. These fields were optional but listed consistently with other demographic data requested of the patient. If the patient did not fill out their demographic data in MyChart, the site used a paper intake form in clinic. Multiple staff members had user rights to update SOGI information in the EHR. Another center (academic; South) had electronic patient questionnaires verified by a clinician. Finally, 1 center (academic; West) that systematically collected SOGI data had a 1-on-1 registration process (Box 2).

Box 2. **Emergent Themes and Additional Exemplary Quotes for SOGI Data Collection**

**CFIR domain: outer setting**

**External mandates: leadership mandate and structured workflows**

“About 3 years ago we became a subsidiary of a large academic institution. And… over the past 3 years… there’s been much more focus on collecting information and trying to be sensitive to gender identification, both in organizational activities and in collecting information.” (Nonacademic; Northeast)

**External mandates: accrediting and quality assurance body**

“Like CMS is coming out with its next iteration of an alternative payment model that’s the Enhancing Oncology Model. And I believe that part of the data collection in there includes not only social determinants of health, but a component of, um, this, this type of data collection… I’ve noticed that like national organizations like Oncology Nursing Society or American Society of Clinical Oncology are really focusing more and more into diversity, inclusion, sexual orientation, and gender identity. It used to be you couldn’t find a whole lot of information and now you’ve got the support of, um, national organizations relaying information and, and giving us the, the push to do it.” (Academic; Midwest)
“ASCO has a report, equity report card, right? We’re not sure what the metrics are yet. We’re still waiting for that report. But the thing is now equity is something for you to be a quality cancer [center].” (Academic; Northeast)

**Market competition**

“We have 2 major hospital systems competing, well, 3 in a very small geographic area. I think that internal competition can drive the C-suite [Chief Executive Officer, etc] to do things they wouldn’t do otherwise.” (Academic; Midwest)

**CFIR domain: inner setting**

**Institutional culture**

“I think it’s just ingrained in our brain to treat everyone like they’re a family member, whether they’re your doctor, your coworker, you know, I think that’s just part of our culture here… So I, I think everybody’s very respectful.” (Nonacademic; Northeast)

**Perception that SOGI data are relevant to clinical practice: education on the importance of SOGI collection**

“It wasn’t until our folks really understood [the value of SOGI data] that they bought into it because it was a struggle getting people to even think it was important to collect.” (Academic; Midwest)

**Patient safety**

“We don’t want them to feel upset or pressured. …If a patient comes to the front desk and they say that their, their name is James and it doesn’t match with what they appear… the front desk person is gonna say… “I see that this doesn’t match with your driver’s license. What kind of a notation would you like me to make in your chart?” (Academic; Midwest)

**CFIR domain: individuals**

**Leadership support: person or people responsible for SOGI data collection**

An urban institution hired a care coordinator to “look at some of that data and then determine if people need to be navigated to other services outside of the cancer center.” (Academic center; Northeast)

**Clinical or administrative champions**

“From a clinical standpoint, I think that there has been a big push on our campus. There are a lot of… clinical researchers kind of trying to work in this space on campus. And I think that’s been, um, that’s been really helpful from a clinical standpoint to make that, this sort of stuff more visible. And that trickles down from like the lead, the leads of our cancer center becoming more supportive of this and more, more, acknowledging that this sort of stuff is very important for clinical care in terms of a day-to-day standpoint, but also from a research standpoint.” (Academic center; Midwest)

**CFIR domain: implementation process**

**Training for SOGI data collection**

“[Training] sessions… were repeated almost every 6 months until we kind of got people to understand the, the importance of it. […] The, the 6-month component of it was truly getting people on board. It took that much time and education to get people on board just to roll it out.” (Academic; Midwest)
“[We] had 2 different trainings… One… was to meet with the, head NP and head physician and… give the background of… why we were focusing on SOGI data collection… We did a physical demonstration of where the information lived in Epic… [Training champions] relied on [the NP and physician leader] to go back and train, um, the other physicians and the other NPs in the practice.” The second training was a “6-session, 15-minute curriculum… delivered… over the course of 6 weeks that went over key terms and concepts… How to ask SOGI questions, why it’s important, what happens when you make a mistake, how to recover from a mistake, and then internal and external resources that people can use if they want more information.” (Academic; Northeast)

**IT systems to capture SOGI data: built systems and patient portals**

“I think in 2016… We were one of the first organizations to do that… Epic at that time had not built sexual orientation gender identity questions. So our IT department really developed those questions.” (Academic; Northeast)
“[T]he health system did like a big email push for the, the folks that are subscribed to the patient portal and, and people can put in their sexual orientation preferred name, preferred pronouns, gender identity directly in that, and then it, it translates directly into the health record and updates.” (Academic center; Midwest)

**Forced workflow: procedural redundancies to optimize completeness of data collection**

“Yeah, so ideal state would be that they do it ahead of time within MyChart to do the pre-check in… it’ll pop up just for them and they’ll, um, again, I think there’s an option where they can, um, like decline to put something in there. Um, but they do, it does pop up for them to, to answer.” (Academic center; Midwest)
“When you go into MyChart, no matter what it, our MyChart system prompts you to update your information and in the information update prompt, which it kind of gives you every time or every so often you log in, that is one of the screens that you go to, and you can either leave it blank or fill it in.” (Nonacademic center; Midwest)

**Forced workflow: 1-on-1 registration process**

“Here at our center we do have a 1-on-1 registration process. They sit down in a private area with us to fill out those forms [that include SOGI questions]. They fill them out on their own. We answer any questions if they have any questions.” (Academic; South)

Abbreviations: ASCO, American Society of Clinical Oncology; CFIR, Consolidation Framework for Implementation Research; CMS, Centers for Medicare & Medicaid Services; IT, information technology; NP, nurse practitioner; SOGI, sexual orientation and gender identity.

A few institutions found that market competition played a role in SOGI data prioritization. One interviewee (academic; Northeast) reported, “I do think she felt a little bit of pressure because of [name] and the other hospitals who were collecting it.” By contrast, at an institution (nonacademic; South) where SOGI data were not collected, “the major competitor in the market is a Catholic organization, so I don’t know that there would be market pressure from there.”

For sites systematically collecting or planning to collect SOGI data, leadership support was important. At 1 site (academic; Northeast), a respondent indicated that “Having leaders who are not part of the community wear pins, have flags in their office, [give] us time at faculty meetings to present… on the SOGI data project” and including it in scorecard metrics were important. For sites partially collecting SOGI data, interviews indicated that support from leadership was critical. Conversely, at sites where SOGI data were not collected, there was minimal mention of leadership support. However, one interviewee (academic; Midwest) indicated that SOGI data collection would have “to start at the leadership level.” Another interviewee from the same institution said, “I wouldn’t say that it is a priority for them to get out in front on issues like this.”

Leadership support went hand in hand with a supportive institutional culture. An interviewee (academic; South) said, “I think that culture and having leaders” was important. “We value [these data] from a cultural, institutional standpoint… because now we can see these and utilize… and hopefully start acting on them.” In contrast, an interviewee (nonacademic; South) said that if their colleagues were polled, “they would kinda give… lip service… but they’re still joking in the background about… deadnaming or… intentional misgendering.”

Clinician and staff comfort varied in asking patients about SOGI data regardless of setting. People implementing change needed to understand why SOGI data collection is relevant to clinical care. One person at an institution that systematically collected SOGI data (academic; Northeast) emphasized the importance of clinicians “realizing… [SOGI] has direct relevance for patient care and is applicable.” In contrast, at sites where SOGI data were not being collected, SOGI data were not perceived as relevant to clinical practice. An interviewee (academic; Midwest; rural) said, “Why would we ask?… It really doesn’t play a role in caring for our patient.” An administrator (academic; South; urban) from a progressive area with a large SGM community said, “I just don’t know the frequency of which… that data would be impactful… would it be 1% of our patients, 10%, 1 in 500?”

Training was critical to reinforce the importance of SOGI data with team members; content included how to collect SOGI data, SGM barriers to care and cancer disparities, relevance to clinical care management, communication strategies for affirming care, and visual displays of support for SGM patients. Some interviewees noted that training on basic terminology would have been helpful early on. Training was more likely to be integrated into physician and staff onboarding processes in settings where SOGI data were systematically collected. To remedy clinician discomfort asking about SOGI, 1 site (academic; Midwest) had “quite a few education sessions. We’ve brought in gay men and physicians… to have them speak to the nurses and social workers [and] even the physicians.”

Less commonly, scripts and environmental cues reinforced training and helped to normalize SOGI data collection. One strategy for normalizing SOGI data collection was using standardized language during patient intake. One center (academic; Northeast) had a script that indicated: “I’m gonna ask these next questions… which we ask all patients… in order to provide good care.” Environmental cues included rainbow flags and signage asserting that everyone deserves equal care. At that same cancer center, an interview reported that “one of our PFAC [Patient Family Advisory Council] members talked about… how meaningful it was to see the signage displayed and to be asked the questions when he was in the visit.”

Early adopters built information technology systems to capture SOGI data (eg, 2 centers had SOGI questions built into Epic). Patient portals were an important tool for data collection, particularly for a site where researchers valued SOGI data collection, but data were not necessarily being used to inform clinical practice. Likewise, 1 center (academic; Midwest) depended on intermittent automated pushes to patients asking them to complete demographic data in the patient portal. Most sites that partially collected SOGI data relied on optional self-report through a patient portal.

Where SOGI data were used to inform practice, information technology supported accountability structures to monitor and improve data collection. An interviewee (academic; Midwest) indicated, “[W]e are tracking the adherence to screening for the SOGI tool.” At another center (nonacademic; Northeast), “[Physician] charts get audited… They get graded on that.” Data systems identifying disparities planned to address them through quality improvement. A center (academic; Northeast) “developed a Tableau (Salesforce Inc) dashboard to look at performance,” and give “results back to the practice managers.”

In Midwestern and Southern settings, building community trust was mentioned as critical before system improvements could be implemented. In Northeastern settings, the community was more often reported as open to SOGI data collection, with the exception of a center that served a very rural area.

For some institutions that systematically collected SOGI data, patient safety was a concern. Conversely, at some institutions that were not collecting SOGI data, there were perceptions of community backlash. One respondent (nonacademic; South) indicated, “You’re gonna get someone at some point that is offended.” See Box 2 for exemplar quotations of constructs identified in the results given.

## Discussion

Past research has shown patient willingness to answer SOGI questions in the context of oncology care.^14^ Barriers to data collection have been associated with lack of perceived relevance of SOGI data to practice, fear of patient discomfort, lack of formal processes for data collection, and limited awareness of electronic fields that capture SOGI data.^6^ Past research has shown that SOGI data collection was associated with leadership support, perceived relevance of SOGI data to practice, resources, structured data fields, patient pronoun querying, geographic location in the Northeast, presence of minority outreach staff, and higher proportion of White populations.^5,15,16^ The present study provides context for many of these prior findings.

Implementation factors relevant to SOGI data collection are multilevel and span the domains of the CFIR. Three of the 7 high-performing sites were in the Northeast, where state laws supported or mandated SOGI data collection and where communities were more likely to expect affirming care environments. A primary factor associated with institutional change was a mandate coming from some level: state, accrediting body, or leadership. For sites without a mandate, systematic SOGI data collection was facilitated by a forced workflow, redundancies in data collection to optimize accuracy and completeness of data capture, or a highly personalized 1-on-1 registration process that included SOGI data collection. The belief that SOGI data would be required in the future by payers and funders also motivated change.

For sites that did collect SOGI data, asking about recorded sex and gender identity as separate fields and capturing name in use, pronouns, and sexual orientation were typically collected. Organ inventories (a tool used to screen for what organs are present in a person and whether any have been removed or altered through surgery, regardless of external sex organs, sex characteristics, or gender identity) were rarely collected, and gender of partner was never collected. Both in the description of how SOGI data importance evolved within institutions and in the process of interviewing, we found that perception of the importance of SOGI data to clinical practice was shaped by exposure to SGM people.

To our knowledge, this study is the largest known study to conduct in-depth interviews about SOGI data collection across diverse oncology practices nationwide. Although the structure, capacity, and clinical flow of individual practices varied widely, we found common facilitators. These qualitative findings add depth to the prior quantitative study.^5^ In addition, respondents from sites that did not collect or use SOGI data indicated that the only way such collection was likely to happen was with a mandate.

The implications of our study are far reaching. Our research affirms the power of a mandate and forced workflows to change institutional practice. Our findings would likely translate across clinical care settings as our results reflected factors that were not oncology specific. Given geopolitical differences in the US at this time, including a precedent of at least 1 institution disclosing SOGI data to state government leaders^17^ and recent executive orders stigmatizing transgender persons,^18^ ensuring safety of patients is paramount. Comprehensive policies that protect individuals from release of information, training for clinicians and staff regarding the importance of SOGI data to health, and enforcement of patient confidentiality are needed.

### Limitations

This study has some limitations. Our sample was subject to social desirability bias; individuals with less interest in SOGI data collection were less likely to respond to interview invitations.

## Conclusions

This qualitative study of implementation factors associated with SOGI data collection in outpatient oncology practices found that systematic SOGI data collection occurred in a few centers. To our knowledge, this is the largest known study to provide qualitative details on implementation catalysts to SOGI data collection in oncology. Future work is needed to identify necessary and sufficient conditions for SOGI data collection using additional approaches.

## Notes

Supplement 1. **eAppendix.** Interview Guide

Supplement 2.
Data Sharing Statement
