# Supplementary material for: Patient Sexual Orientation and Gender Identity Information Practices in Oncology
Source: JAMA Netw Open. 2025 Jun 23;8(6):e2516941. doi: 10.1001/jamanetworkopen.2025.16941 (PMC12186564; doi:10.1001/jamanetworkopen.2025.16941)
Supplement: Supplement 1. — eAppendix. Interview Guide [file jamanetwopen-e2516941-s001.pdf]

Supplementary Online Content

Pratt-Chapman ML, Mullins MA, Gold B, et al. Patient sexual orientation and gender identity information practices in oncology. *JAMA Netw Open.* 2025;8(6):e2516941. doi:10.1001/jamanetworkopen.2025.16941

eAppendix. Interview Guide

This supplementary material has been provided by the authors to give readers additional information about their work.

© 2025 Pratt-Chapman ML et al. *JAMA Network Open.*

eAppendix. Interview Guide

Date: ________________
Time: ________________
Interviewer: ________________
Interviewee Study ID: ________________
Notetaker: ________________

Intervention: SOGI data collection and use

1. Tell me about your role at your institution and how it relates to demographic data collection and use.
2. Tell me about any data related to sexual orientation, behaviors, relationships, gender, etc. that are collected in your organization.

   As a reminder, please do not use your institution's name or any names of colleagues to optimize anonymity of these data. Feel free to mention professional roles.

   Probe for:

   - Wording of question stems and response options for SOGI items being used
   - Sex assigned at birth
   - Gender identity of patient
   - Two-question process used to collect sex and gender
   - Patient pronouns
   - Patient's name in use if it differs from their legal name
   - Prominently display of name in use / banner or a pop-up easily accessible for front line staff and providers
   - Patient's organ inventory
   - Sexual orientation if patient chooses to provide it
   - Explicit options for recording parents are inclusive of same-sex parents and other diverse families [if pediatric]
   - Relationship status, including with an un-married partner

© 2025 Pratt-Chapman ML et al. *JAMA Network Open.*

- Gender identity of partner [when applicable]

3. Where in the EHR are these elements documented?
    - Structured fields
    - Unstructured fields
    - Scanned notes (probe for: by whom?)

   If not in the EHR, are these data captured in any other way? If so, where/ how?

Process/ Individuals

4. Walk me through the process of how any SOGI type data may be collected at your center.

   How are these data collected? By whom?

   Probe for:
    - Patient-initiated discussion
    - Collected by front desk? E.g., via intake form
    - Patient portal? Electronically? Before apptmt? After apptmt?
    - By clinician? What type? How? E.g., nurse or other HCP asks verbally
    - Who ensures these data are documented in the EHR?
5. How consistently or systematically are these data collected?
    - Systematic/ Part of routine intake?
    - Ad hoc? Random?
    - If patient discloses?
    - How often are these data collected? Once, or at every visit?
6. How are SOGI data used in your practice? (probe for below)
    - PROBE: What does “use” mean to the interviewee?
    - PROBE: If the interviewee gives examples, e.g., “to improve communication” or “inform medical management,” ask what that means to interviewee
7. Tell me about any barriers to collecting and using SOGI data.

© 2025 Pratt-Chapman ML et al. *JAMA Network Open.*

- EHR? / IT?
- Time?
- Resources needs?
- Org culture?/ Inertia/ Lack of buy-in?

8. Tell me about anything that has helped you and your organization systematically collect and use SOGI data.
    - EHR ready-to-use modules?
    - IT availability to support optimization of EHR
    - Training?
    - Allocated resources?
    - Culture of Quality Improvement?
    - Clinical/ Administrative Champions?
  - How can current processes be improved? OR: If your organization does not currently collect SOGI data, what do you think the best way to systematize SOGI data collection would be?

Inner setting/ Individuals

9. How comfortable and capable do you feel about collecting SOGI data?
    - Interviewee comfort
    - Self-efficacy
10. What kind of reactions have you experienced when patients are asked about SOGI?
    - From LGB patients
    - From patients of trans/nonbinary experience
    - From het/cis patients
    - Do patients have positive, negative, neutral reactions to being asked about SOGI?
        - Differences based on age of patients or other demographics?

© 2025 Pratt-Chapman ML et al. *JAMA Network Open.*

11. Tell me about your organizational culture and how it relates to collection and use of SOGI
    - Organizational readiness to collect/ use SOGI data
    - Prioritization of systematically collecting SOGI data
    - Internal incentives
        - Goal sharing
        - Use of data collection fidelity in performance feedback?
        - Communication channels or IT that reinforces data collection
        - Recognition / engagement / support from leadership
        - Appreciation from patients?
    - Organizational mission, norms, values
    - Clinician buy-in? / Champions? / Role models
        - Belief in relative advantage/ utility of SOGI
    - Staff perceptions: Belief in relative advantage/ utility of SOGI
    - Culture of learning / quality improvement?
12. Has your organization provided any types of training that are specific to SOGI data or SGM patient needs?
    - How to collect, and record gender identity data?
    - LGBTQ status as confidential patient information in accordance with HIPAA protections
    - Implicit bias training/bystander training
    - SGM specific cancer risk reduction, screening, treatment considerations?
    - For whom?
    - When?
    - What format? Dosage?

Outer setting

© 2025 Pratt-Chapman ML et al. *JAMA Network Open.*

13. Are there any external forces, like pressure from other groups, other healthcare organizations, state or local governments, or the community, that have helped or hindered your SOGI data collection and use?
    - Mandates for meaningful use / Payer considerations?
    - Legislation (local/state/federal)
    - EHR upgrades (eg. EPIC Sex | Gender | Name module)
    - Market competition?

Other comments

14. [For those that collect at least some data.] To summarize, overall what have been the key factors that have motivated your practice/institution to collect the SOGI data that it does?
15. What factors do you think would motivate your practice/institution to increase/change/modify/improve its processes related to collection?
16. Is there anything else you'd like to share about SOGI data collection or any related topics?

If SGM-identifying

17. Do you feel that being someone who identifies as a sexual and/or gender minority has affected your interaction with colleagues or patients in any way? If so how?

© 2025 Pratt-Chapman ML et al. *JAMA Network Open.*
