# Supplementary material for: Patient Sexual Orientation and Gender Identity Information Practices in Oncology
Source: JAMA Netw Open. 2025 Jun 23;8(6):e2516941. doi: 10.1001/jamanetworkopen.2025.16941 (PMC12186564; doi:10.1001/jamanetworkopen.2025.16941)
Supplement: Supplement 2. — Data Sharing Statement [file jamanetwopen-e2516941-s002.pdf]

Data Sharing Statement

Pratt-Chapman. Patient Sexual Orientation and Gender Identity Information Practices in Oncology. *JAMA Netw Open*. Published June 23, 2025.
doi:10.1001/jamanetworkopen.2025.16941

Data
Data available: Yes
Data types: Deidentified participant data
How to access data: Contact the corresponding author.
When available: With publication

Supporting Documents
Document types: None

Additional Information
Who can access the data: Researchers wishing to use data for noncommercial purposes with an approved IRB.
Types of analyses: Noncommercial purposes.
Mechanisms of data availability: With signed data access agreement.
